# Self-Stigma and Quality of Life in Patients With Depressive Disorder in Psychiatric Outpatient Setting

**DOI:** 10.1192/bjo.2024.269

**Published:** 2024-08-01

**Authors:** Chun Wah Wan

**Affiliations:** Kwai Chung Hospital, Kwai Chung, Hong Kong

## Abstract

**Aims:**

Self-stigma is common among patients suffering from depressive disorders and negatively affects their quality of life. Quality of life reflects individuals' general well-being, an important measure of treatment outcomes. However, local research on the relationship between self-stigma and quality of life in patients with depressive disorder is lacking. Information on clinical and personal characteristics associated with self-stigma in depression is also limited.

The primary aim of this cross-sectional study was to examine the relationship between self-stigma and the quality of life of patients suffering from depressive disorder in an outpatient department. The secondary aim was to identify socio-demographic, clinical, or personal characteristics associated with self-stigma in these patients.

**Methods:**

One hundred and thirty-one patients with depressive disorders were recruited from the outpatient clinic of a psychiatric centre in Hong Kong. Depressive disorder was diagnosed with the Chinese-bilingual version of the Structured Clinical Interview for the Diagnostic and Statistical Manual of Mental Disorders, Fourth Edition, Axis I Disorders. Socio-demographic and clinical information were obtained. Self-stigma was measured with the Self-Stigma Scale-Short Form. The quality of life was evaluated with the World Health Organization Quality of Life-BREF Hong Kong Version. Self-esteem, coping strategies, personality traits, and social functioning were evaluated. Bivariate analyses were performed to explore the association between the above factors with self-stigma or quality of life. Regression analyses were conducted to explore the relationship between self-stigma and quality of life, and to identify the factors independently associated with self-stigma.

**Results:**

Self-stigma was independently associated with the four main quality of life domains after controlling for socio-demographic, clinical, and personal characteristics among patients with depressive disorder. A multiple regression model showed that high levels of neuroticism and low self-esteem were independently associated with higher levels of self-stigma.

**Conclusion:**

This cross-sectional study supported the negative association between self-stigma and quality of life among individuals with depressive disorder. Neuroticism and self-esteem were found to be independently associated with self-stigma in depressive patients. Considering the associations found, identifying and focusing on depressive patients with a higher risk of self-stigma and implementing self-stigma interventions is important. Specific self-stigma reduction strategies should be introduced to mitigate the self-stigma in depressive patients and to improve their quality of life.

